# Perceived stigma and the role of BMI on perceived HIV-related stigma among people living with HIV/AIDS in Southeast Ethiopia

**DOI:** 10.3389/fpsyt.2025.1404896

**Published:** 2025-07-28

**Authors:** Fikreab Desta, Demisu Zenbaba, Biniyam Sahiledengle, Shifera Metaferia, Tesfaye Desalegn, Degefa Gomora, Chala Kene, Girma Beressa, Telila Mesfin, Pammla Petruka, Lillian Mwanri

**Affiliations:** ^1^ Department of Public Health, Goba Referral Hospital, Madda Walabu University, Bale Goba, Ethiopia; ^2^ Department of Laboratory, Goba Referral Hospital, Madda Walabu University, Bale Goba, Ethiopia; ^3^ Department of Pharmacy, Goba Referral Hospital, Madda Walabu University, Bale Goba, Ethiopia; ^4^ Department of Midwifery, Goba Referral Hospital, Madda Walabu University, Bale Goba, Ethiopia; ^5^ School of Medicine, Goba Referral Hospital, Madda Walabu University, Bale Goba, Ethiopia; ^6^ Nursing Education, University Saskatchewan College of Nursing, Saskatoon, SK, Canada; ^7^ Research Centre for Public Health, Equity and Human Flourishing, Torrens University Australia, Adelaide, SA, Australia

**Keywords:** perceived HIV-related stigma, HIV/AIDS, Ethiopia, PLWHIV, depression

## Abstract

**Background:**

People living with HIV/AIDS are at an increased risk of perceived HIV-related stigma. The effectiveness of social support for perceived HIV-related stigma is hampered by high depression. Although there is evidence that being underweight is associated with perceived HIV-related stigma, the mechanism is not well known. This study aimed to assess perceived HIV-related and the role of body mass index (BMI) on perceived HIV-related stigma in Southeast Ethiopia.

**Methods:**

A hospital-based cross-sectional study design was conducted among 547 randomly selected HIV/AIDS patients in Southeast Ethiopia. Perceived HIV-related stigma was assessed using a 10-item perceived HIV stigma scale assessment tool. Descriptive statistics were computed, and the data were analyzed by logistic regression, correlation, and mediation model.

**Results:**

The magnitude of perceived HIV-related stigma was found to be 68% [95% CI: (64.1%, 71.9%)] among participants. Patients with low social support [AOR=1.5, 95% CI: (1.05, 2.40)], a body mass index (BMI) of <18.5 kg/m^2^ (kilogram per meter squared) [(AOR = 5, 95% CI: (2.30, 11.0)], and non-adherence to highly active antiretroviral therapy (HAART) [(AOR: 5, 95% CI: (1.03, 3.05)] were significantly associated with perceived HIV-related stigma. In mediation, the results indicated that the total mediation effect (*B* = -0.62, 95% CI [-0.828, 0.404]), direct effect (*B* = -0.30, 95% CI [-0.554, -0.046]), and depression played a chain mediating role (indirect effect) (*B* = -0.41, 95% CI [-0.557, -0.261]) were significant.

**Conclusion:**

The prevalence of perceived HIV-related stigma was found high. Patients with poor social support and non-adherent to HAART were more likely to suffer from HIV-related perceived stigma. Our findings suggest that there is a relationship between body mass index and perceived HIV-related stigma, while depression can indirectly predict perceived HIV-related stigma.

## Introduction

HIV/AIDS is a major public health concern across the world, particularly in low- and middle-income countries (LMICs) (1). More than 74.9 million people worldwide have been infected with HIV (2). Sub-Saharan Africa (SSA), the most affected region, is home to 76% PLWHIV (3). HIV/AIDS-related stigma is seen as prejudice, discounting, ridiculing, and discrimination aimed against those who are suspected of having HIV/AIDS (4, 5).

Perceived stigma describes how people living with HIV (PLWHIV) feel or fear when they are being treated unfavorably (6, 7). The United Nations Programme on HIV/AIDS (UNAIDS) report indicated that over 50% of people globally experience discriminatory attitudes because of their HIV status (8). A study conducted in the United States reported that 89% of PLWHA in the US experienced perceived stigma (9). A study conducted in Botswana and Venezuela reported stigma as a major obstacle to HIV testing (10, 11). The perceived stigma has a significant impact on the quality of life in PLWHIV (12). Stigma limits PLWHIV’s access to care, which is a significant contributor to the global HIV pandemic (12–14).

Different studies conducted in Brazil (15), USA (16), South Africa (17), Zambia (18), and Southern Ethiopia (19) indicated an association between perceived HIV-related stigma and low birth weight. Other studies also revealed that a low BMI significantly increases the risk of developing antiretroviral drug-related liver injury (20) and tuberculosis (21) among people living with HIV patients. The findings from various studies highlight the importance of addressing perceived HIV-related stigma to improve the health outcomes for PWH, particularly in those with low BMI (22).

The national prevalence rate of HIV/AIDS in Ethiopia is 0.9% (23), and the number of PLWHA per region contributes to the varied prevalence rates (24). The Ethiopian Demographic and Health Survey (DHS) 2016 reported a low prevalence of HIV/AIDS (0.7%) in Oromia region (24) within and across nations. The low HIV prevalence may contribute to increased stigma toward PLWHA (25, 26).

In Ethiopia, about 16%–56% of PLWHA reported having perceived HIV-related stigma (27, 28). A stigma index survey done by networks of HIV-positive people in Ethiopia indicated that stigma is acquired and perpetuated through gossip, verbal insult, isolation, and rejection (6).

A recent systematic review indicated that an evidence-based effective programming to reduce stigmatizing and discriminatory attitudes has increased significantly (6, 29). However, money countries has not made reducing stigma a top priority in their national AIDS policies or programs (30), and HIV-related stigma continues to play a significant role in contributing to the spread of the epidemic (31). Although a substantial number of PLWHA live within the study area, the issue of HIV-related stigma has not been well addressed.

Therefore, this study primarily aimed at exploring BMI and its related mechanisms, which are essential to developing mental health interventions, which are becoming increasingly important to improve the mental and physical quality of life among HIV/AIDS patients. However, to the best of our knowledge, no studies have explored how and when BMI affects perceived HIV-related stigma in PLWHIV. Thus, this study also helps to determine whether depression mediates the association between BMI and perceived HIV-related stigma.

## Methods

### Study design and setting

A hospital-based cross-sectional study design was used to assess HIV-related perceived stigma and its associated factors among PLWHIV who receive treatment at antiretroviral therapy (ART) clinics. The Bale Zone is located approximately 412 kilometer (km) away from Addis Ababa. It has three government hospitals, one referral (Goba Referral Hospital), and two general hospitals (Dellomena and Robe General Hospitals) that are currently providing ART services in the Zone. During the period between February 1 and April 30, 2021, there were 3,308 adult HIV/AIDS patients who had registered for ART follow-up in these three public hospitals in the study hospitals.

### Study population

All HIV-positive patients aged >18 years who were enrolled to receive ART treatment follow-up in public hospitals in Bale Zone were the source population (32). The potential participants were randomly selected for inclusion in the study if they had been enrolled for ART for at least 6 months. Patients who were unable to communicate or had a serious medical condition were excluded.

### Sample size determination and sampling technique

The sample size was determined using the single population proportion formula using EPI info version 7.2 assuming the following parameters: 95% level of confidence, 4% marginal error, and 49.4% proportion of HIV-related perceived stigma among PLWHA (4). Moreover, 10% of the potential participants were added to address the non-response rate yielding the total sample size as 559. The study participants were chosen using systematic sampling techniques. The sampling interval was calculated by dividing the total number of patients on ART (*N* = 3 308) by the total desired sample size of 559. As a result, the k number was six, and the fourth patient was chosen at random from the first six ART patients, and then every sixth patient was included in the study. The sample size was allocated proportionally to each hospital after obtaining lists of potential participants from the ART registers of each hospital.

### Variables of the study

#### Dependent variable

The dependent variable is perceived HIV-related stigma.

#### Independent variables

The socio-demographic variables included (age, sex, religion, residence, marital status, education level, occupation, and monthly income). The psychosocial variables included living condition, social support, and lost job. Clinically related data were included such as WHO HIV/AIDS stage, current CD4 count, medication adherence, drug regimen, current drug side effect, duration of HAART treatment, and viral load.

### Data collection, measurement, procedures, and quality control

A data collection tool (questionnaire) that included socio-demographic, psychosocial, and disease-related information was developed after reviewing relevant literatures. The questionnaire was originally developed in the English language and translated into Amharic and local language (Afaan Oromo) by language experts. The Amharic and Afaan Oromo version was translated back to English to verify the consistency by language experts. Both of the Amharic and the Afaan Oromo language questionnaires were used to collect data.

HIV-related perceived stigma occurs when PLWHA feel/perceive or believe that they are being negatively treated by others including partners, family, friends, healthcare providers, and members of their community because of their HIV status (6). Perceived stigma was assessed using the HIV-related stigma scale assessment tool which contains 10 stigma assessment questions with Likert scale. The agreement questions (strongly disagree–strongly agree) were assigned values 1–5 in order to determine the level of perceived stigma. Respondents who scored higher than the mean from the total were considered to have experienced perceived stigma, whereas those who scored lower than the mean were considered to have not experienced perceived stigma (33, 34).

Additionally, social support was assessed using the Social Support Questionnaire-6 (SSQ-6) which assessed the available social support (35). It had six assessment questions (help from no one, help from family, friends, organization, regions father/person, and unknown persons); those respondents who scored higher than the mean were considered to have good social support, while those who scored lower than the mean were considered to have poor social support. Thirdly, the structured Patient Health Questionnaires-9 (PHQ-9) was used to measure the depression status of HIV/AIDS patients (36). It had a potential total sum score of 27 from nine items; those respondents who scored 5 and above in the total sum were considered depressed, while those scoring below 5 were considered non-depressed. Fourthly, the adherence was assessed using the Morisky Medication adherence scale questions eight (MMAS-8) that had a total sum score of 8 from eight items (37, 38). Respondents who scored below six were considered non-adherent, while those who scored six and above were considered adherent.

Data were collected using interviewer-administered questionnaires and by extracting pertinent information from the patients’ medical records. Four data collectors with bachelor of science (BSc) degrees prepared; nurses, midwifes, and public health were involved in data collection. A one-day intensive training was given on the objective of the study, how to fill the questionnaire, confidentiality of the information, and interviewing technique prior to their involvement for data collection. A pretesting of the questionnaire was done on 5% of the total sample size in a non-study area, and appropriate amendments were made before the actual data collection. The completed questionnaires were reviewed and checked for completeness, consistency, and relevance daily.

### Data processing and analysis

The data were entered into Epi Data™ version 3.1 before being exported to Statistical Package for Social Science (SPSS ™) version 25 for cleaning, coding, and analysis. Descriptive statistics such as frequency, percentage, and mean were computed and presented by using text, tables, and graphs. Bivariable binary logistic regression was undertaken to see the association between dependent and independent variables. Those variables having a *p*-value of <0.25 in bivariable binary logistic regression were included in the multivariable logistic regression model. Both crude odds ratio (COR) and adjusted odds ratio along with 95% confidence interval (CI) were used to estimate the strength of the association between factors and outcome variable. In the multivariable logistic regression model, variables having a *P*-value of <0.05 were considered statistically significant. The Hosmer and Lemeshow test was used to determine the final model’s fitness, and the variance inflation factor was used to check for multi-collinearity among selected independent variables. We also used a mediation model in which body mass index served as the independent variable and perceived HIV-related stigma served as the dependent variable. In the model, depression was used as a mediating variable. Based on 5,000 bootstrap samples, the chain-mediating effect was estimated using the bootstrap 95% confidence interval (CI). All covariates were taken into account in the analysis.

## Results

### Socio-demographic and economic characteristics of study participants

A total of 547 study participants were included in the study, yielding an overall response rate of 97.8%. The respondents’ mean age (+SD) was 38.1 (± 9.8) years. A total of 274 (50.1%) respondents were male, 340 (62.2%) were married, and 200 (36.6%) were between the ages of 29 and 39 years. Almost one-third, 164 (30.0%), of the respondents attended primary education, 240 (43.9%) were Orthodox religion followers, 126 (23.0%) were housewives, and 402 (73.5%) lived in urban areas (**Table 1**). This study also identifies a negative association between body mass index and perceived HIV-related stigma in PLWHIV.

**Table 1:** Socio-demographic and economic characteristics of people living with HIV/AIDS in public hospitals in Southeast Ethiopia, 2021 (*n* = 547).

Variables	Frequency	Percent
Sex		
Male	274	50.1
Female	273	49.9
Age category		
18–29	105	19.2
30–39	200	36.6
40–49	184	33.6
≥50	58	10.6
Residence		
Urban	402	73.5
Rural	145	26.5
Ethnicity		
Oromo	360	65.8
Amhara	164	30.0
Others*	23	4.2
Religion		
Protestant	91	16.6
Orthodox	240	43.9
Muslims	197	36.0
Catholic	16	2.9
Others**	3	0.5
Education status		
Unable to read and write	60	11.0
Able to read and write	104	19.0
Primary school	164	30.0
Secondary or preparatory	159	29.1
College and University	60	11.0
Marital status		
Single	67	12.2
Married	340	62.2
Widowed	73	13.3
Divorced	67	12.2
Occupation		
Farmer	126	23.0
Housewife	126	23.0
Government employee	114	20.8
Daily laborer	113	20.7
Others***	68	12.4
Income level		
≤500	94	17.2
501–1,500	133	24.3
1,501–2,500	119	21.8
2,501–3,500	80	14.6
≥3,501	121	22.1
Family size		
<5	448	81.9
5–7	79	14.4
≥8	20	3.7

*Dawuro, Woliyta.

**Jehovah’s witness, Adventists, woqefeta.

***Merchants, non-governmental employee.

### Psychosocial and clinical-related characteristics of respondents

Of the 547 study participants, 437 (79.9%) lived with their families, 153 (28%) lost their jobs due to HIV/AIDS-related illness, and 375 (68.6%) received inadequate social support from their families or other supportive bodies. Most of the respondents, 434 (79.3%), were at WHO clinical stage I, whereas 12.4% were at WHO clinical stage II. About one-third [181(33.1%)] of the respondents had a CD4 count greater than 500 cells/μL. More than two-thirds of 442 (80.8%) respondents had good adherence to HAART. Of all the study participants, 151 (27.6%) had HAART-related side effects, 441 (80.6%) were on the first line of the drug, and 503 (92%) were on HAART for more than or equal to 2 months (**Table 2**).

**Table 2:** Psychosocial and clinical characteristics of people living with HIV/AIDS in public hospitals in Southeast Ethiopia, 2021 (***n*** = 547).

Variables	Frequency	Percent
Living condition		
Alone	89	16.3
Live with my families (like wife, mother, father, sister, brother)	437	79.9
Others*	21	3.8
Lost job due to HIV		
Yes	153	28.0
No	394	72.0
Opportunistic disease		
Do not have	469	85.7
Toxoplasma	17	3.1
Fungus	21	3.8
Tuberculosis	40	7.3
BMI category		
<18.5	112	20.5
18.5–24.99	390	71.3
25–29.99	45	8.2
Source of infection		
Blood contact	97	17.7
Unsafesexual intercourse	213	38.9
I do not know	237	43.3
Comorbidity		
No	482	88.1
Diabetes mellitus	26	4.8
Hypertension	39	7.1
Substance use		
Khat	87	15.9
Alcohol	135	24.7
Cigarettes	40	7.3
I do not use	285	52.1
Social support		
Poor social support	375	68.6
Good social support	172	31.4
Depression status		
Depressed	376	68.7
Non depressed	171	31.3
WHO clinical stage		
Stage I	434	79.3
Stage II	68	12.4
Stage III	45	8.2
CD4 count current		
<200	59	10.8
200–349	131	23.9
350–499	176	32.2
≥500	181	33.1
Adherence to medication		
Poor adherent	442	80.8
Good adherent	105	19.2
Drug side effect		
Yes	151	27.6
No	396	72.4
Drug regimen		
First line	441	80.6
Second line	106	19.4
Duration on HAART (in month)		
12	16	2.9
13-24	28	5.1
≥25	503	92.0

BMI, body mass index; HAART, highly active anti-retroviral therapy; CD4, cluster of differentiation four.

*Living with close friends.

### Prevalence of perceived HIV-related stigma

**Figure 1:**
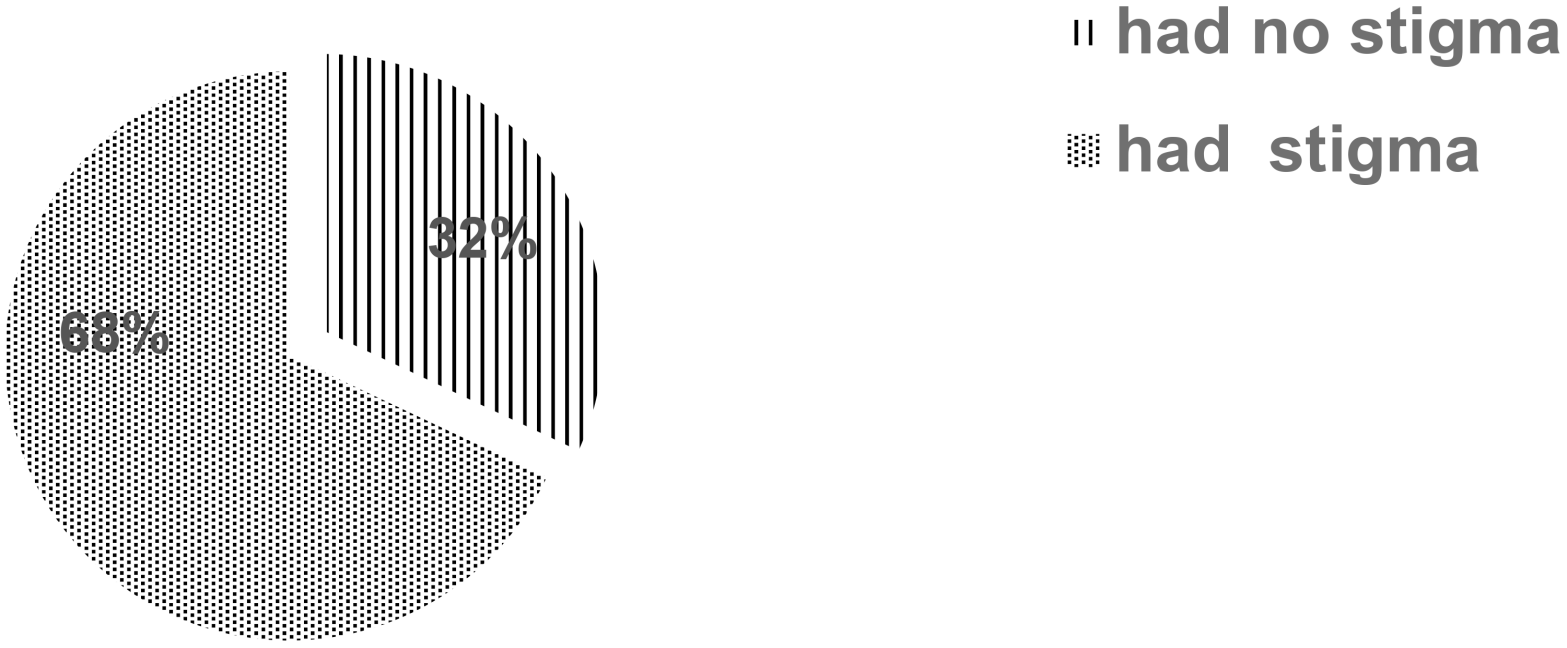
Prevalence of perceived HIV-related stigma among people living with HIV/AIDS at hospitals in Southeast Ethiopia.

The prevalence of perceived HIV-related stigma among people living AIDS was found to be 68% [95% CI: (64.1%, 71.9%)] (**Figure 1**).

### Factors associated with HIV-related perceived stigma

The multivariable binary logistic regression analysis result showed that having a BMI <18.5 kg/m^2^ [AOR = 5, 95% CI: (2.3, 11.0)], BMI 18.5–24.99 kg/m^2^ [AOR = 3.6, 5, 95%: (1.88, 7.16), poor social support [AOR = 1.5, 95% CI: (1.05, 2.40)], and non-adherent to HAART [AOR=1.7, 95% CI: (1.03, 3.05)] were significantly associated with perceived HIV-related stigma (**Table 3**).

**Table 3:** Model for perceived HIV related-stigma and predictors among people living with HIV/AIDS in public hospitals of Bale Zone, Southeast Ethiopia, 2022 (*n* = 547).

Variables	Category	Stigma		COR (95% CI)	AOR (95% CI)
		No	Yes		
Sex	Male	81	193	1	1
	Female	94	179	0.79 (0.55, 1.15)	0.71 (0.46, 1.1)
Age	18–29	33	84	1	1
	30–39	58	130	0.88 (0.52,1.46)	0.93 (0.52, 1.65)
	40–49	61	109	0.70 (0.42,1.17)	0.66 (0.36, 1.20)
	≥50	23	49	0.44 (0.44, 1.58)	0.83 (0.39, 1.77)
Marital status	Single	21	46	1	1
	Married	10	236	1.03 (0.58, 1.82)	1.2 (0.62, 2.25)
	Widowed	30	43	0.65 (0.32, 1.31)	0.77 (0.33, 1.78)
	Divorced	20	47	1.07 (0.51, 2.23)	1.4 (0.60,3.25)
Substance use	Khat	30	57	0.89 (0.53, 1.4)	0.76 (0.42, 1.35)
	Alcohol	40	95	1.1 (0.71,1.73)	0.94 (0.57, 1.55)
	Cigarette	14	26	0.87 (0.43,1.74)	0.83 (0.37,1.84)
	Do not use	91	194	1	1
HIV stages	Stage I	125	309	1.8 (0.96,3.38)	1.78 (0.88, 3.58)
	Stage II	31	37	0.87 (0.40,1.86)	0.89 (0.39, 2.04)
	Stage III	19	26	1	1
CD4 counts	<200	18	41	1.24 (0.66, 2.34)	1.1 (0.54, 2.05)
	200–349	37	94	1.38 (0.85, 2.26)	1.4 (0.83, 2.43)
	350–499	56	120	1.17 (0.75, 1.8)	1.2 (0.75, 1.93)
	≥500	64	117	1	1
BMI (kg/m^2^)	<18.5	27	85	5 (2.46, 10.89)*	5 (2.30, 11.0)*
	18.5–24.99	120	270	3.7 (1.95, 7.01)*	3.6 (1.88, 7.16)*
	>25	28	17	1	1
Level of depression	Depressed	130	246	1	1
	Non-depressed	45	16	1.4 (0.99, 2.21)	1.5 (0.96, 2.35)
Social support	Poor social support	109	266	1.5 (1.03, 2.22)*	1.5 (1.05, 2.40)*
	Good social support	66	106	1	**1**
HAART adherence	Adherent	147	295	1	**1**
	Non-adherent	28	77	1.3 (0.85, 2.2)	1.7 (1.03, 3.05)*

*p-value < 0.05.

BMI, body mass index; COR, crude odd ratio; AOR, adjusted odd ratio; HAART, highly active anti-retroviral therapy.

### Correlation analysis of BMI, stigma, and depression

**Table 4** shows Pearson partial correlations among key variables after controlling for all covariates. All key variables were significantly associated. BMI was negatively associated with stigma (*r* = -0.196, *P* < 0.001). Stigma was positively associated with depression (*r* = 0.518, *P* < 0.001)., 

**Table 4:** Correlations matrix between stigma, body mass index, and depression.

	Mean (SD)	Stigma	BMICAT	Depression
Stigma	30.07 (9.4)	–		
BMI	20.98 (2.78)	-0.196***	–	
Depression	9.24 (7.13)	0.518***	-0.24***	–

All correlations were controlled for age, sex, education level, marital status, income, CD4 count, viral load, and time since HIV diagnosis.

BMI, body mass index.

***P < 0.001.

### Mediating roles of depression

**Table 5** shows the mediating roles of depression in the association between body mass index and perceived HIV-related stigma.

**Table 5:** The mediating roles of depression.

	Effect size	SE	Boot LLCI	Boot ULCI	*P* > |*z*|
Direct effect	-0.30	0.129	-0.554513	-0.045527	0.021
Indirect effect	-0.41	0.076	-0.557186	-0.261132	0.000
Total effect	-0.71	0.108	-0.827837	-0.403956	0.000

A model analysis was adjusted to age, sex, CD4 count, marital status, viral load, educational level, social support, and adherence to ART.

Indirect effect: body mass index → perceived HIV-related stigma

Indirect effect: body mass index → depression→perceived HIV-related stigma

Total effect: body mass index → perceived HIV-related stigma

## Discussion

Internationally, it has been acknowledged that stigma kills more people than the HIV virus itself (39). The study attempted to ascertain the prevalence of HIV-related perceived stigma and associated factors among HIV patients attending an anti-retroviral treatment follow-up clinic at public hospitals of Southeast Ethiopia. The finding showed that the prevalence of HIV-related perceived stigma was high (68%) among the participants. Variables like BMI <18.5 kg/m^2^, BMI 18.5–24.99 kg/m^2^, poor social support, and non-adherence to HAART were significantly associated with perceived HIV-related stigma.

This study revealed that the prevalence of perceived stigma was higher compared to studies conducted in the hospitals of Oromia and Dessie city health facilities in Ethiopia (28, 40) and Cameron (13); however, it was lower than studies conducted at Jimma in Ethiopia (6), Iran (12), and University of Washington (41). This discrepancy might be explained by time variation and differences in setting, particularly for research conducted outside of Ethiopia. It might also be attributed to various governmental and non-governmental efforts in the respective sites.

In the current study, PLWHIV with a BMI of less than 18.5kg/m^2^ (underweight) were five times more likely to have perceived HIV-related stigma as compared to their counterparts. In the past, being underweight was used as a diagnostic criterion for HIV infection (42); hence, PLWHIV might be worrying about their body weight because as they become thinner or lose bodyweight they feel or fear discrimination.

In this study, social support was significantly associated with the level of perceived HIV-related stigma. Study participants with poor social support were 1.5 times more likely than their counter-parts to experience perceived stigma. However, there is evidence that social care and support should be provided in accordance with suitable social and ethical procedures rather than having PLWHIV being perceived as a distinct individual from the rest of the community sponsored by NGOs, which may result in significant stigma (6). Further investigation is needed to ameliorate this issue.

Despite this, the provision of ART was observed to increase the lifespan of people living with HIV/ADIS. Discrimination and a high burden of HIV/AIDS-related stigma, which is induced by socio-demographic, psychological, behavioral, and clinical variables, jeopardize this longer lifetime (43). This study also revealed that respondents who were non-adherent to HAART were 1.7 times more likely to have perceived HIV-related stigma. This could be due to stigma and discrimination, fear of being found, lack of social support, and poor health outcomes that may all play a role in emotional non-adjustment to HIV/AIDS, depression, and a loss of motivation in treatment; this would ultimately lead to poor treatment adherence among PLWHIV (44).

**Figure 2:**
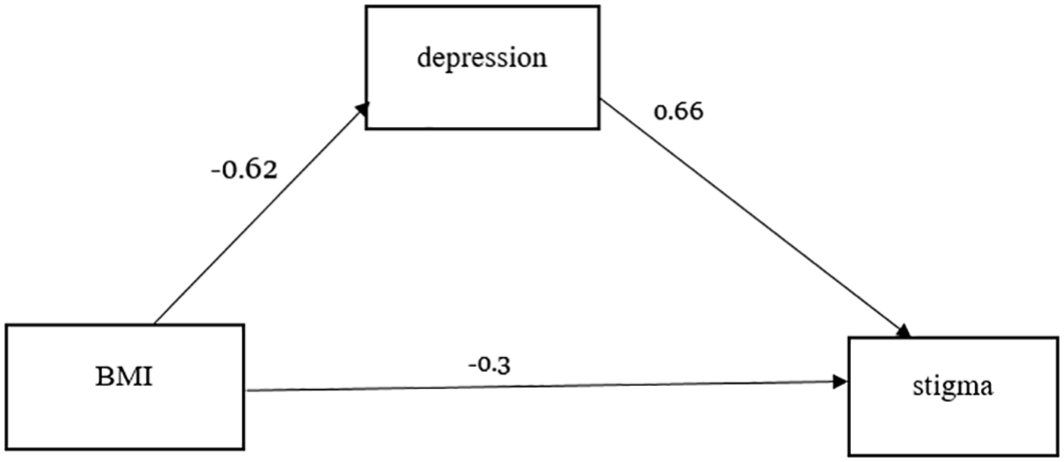
Specific path of the associations between body mass index and perceived HIV-related stigma (mediating effect).

This study revealed a negative association between body mass index and perceived HIV-related stigma in PLWHIV (**Figure 2**). More importantly, the findings of this study suggest important roles for psychological status (depression) in the reduction of perceived HIV-related stigma in PLWHIV by body mass index. According to current studies, PLWHIV with a low body mass index (<18.5 kg/m^2^) (42) experience more severe perceived HIV-related stigma because of higher depression.

This study suggests that body mass index affects perceived HIV-related stigma in PLWHIV with high depression (45), which adds numerical evidence to how body mass index affects perceived HIV-related stigma. PLWHIV who have a normal body mass index (18.5–24.9 kg/m^2^) might not worry about their body weight, and they are not feeling or fearing discrimination. These all contribute to a reduction in depression and thus a reduced risk of perceived HIV-related stigma. Thus, the study indicates that depression can be considered a potential psychological mechanism underlying the relationship between body mass index and perceived HIV-related stigma. Future researchers better explore other variables’ roles in this association.

### Limitation of the study

In a study area lacking previous data on the prevalence and risk factors associated with HIV/AIDS, the aim of this study was to investigate the role of BMI on perceived HIV-related stigma. The following limitations must be taken into consideration when interpreting the study’s results, even though it employed primary data on the extent of perceived HIV-related stigma, depression, and social support with skilled data collectors and supervisors: First, a cause-and-effect relationship between the risk factors and perceived HIV-related stigma cannot be established due to the cross-sectional nature of the study. Second, self-reported surveys of social support, depression, and perceived stigma were used to evaluate the participants; however, these surveys may be liable to social desirability bias. Thirdly, because the study is centered in a hospital, the finding may not be generalizable to the total population.

## Conclusion

This study showed that perceived HIV-related stigma was higher among people living with HIV/AIDS attending hospitals in Southeast Ethiopia due to low awareness of the impact of stigma. It is evident that PLWHIV can be stigmatized in a variety of ways, impacting treatment adherence and the mental health of patients living with HIV (46). As such, in order to address HIV management issues, in general, the impact of HIV-related stigma must be addressed with the same effort as vaccine trials and treatment development initiatives. The authors suggest that in all ART clinics, non-payment of services and free therapy should include education to increase awareness on the impact of stigma. The impact of HIV-related stigma including delayed testing, non-disclosure of HIV status, service access barriers, reduced treatment adherence, poor social support, and directly to the mental health among affected individuals and their families are detrimental to global efforts against HIV so far. In addition to addressing stigma in Ethiopia, the clinics should continue the promotion of HAART adherence to sustain people who are already affected by HIV. Our findings suggest that there is a relationship between body mass index and perceived HIV-related stigma, while depression can indirectly predict perceived HIV-related stigma. Importantly, the lessons learned from the findings of the current study need to be considered in the design and implementation of HIV initiatives, including policy making and programming in Ethiopia and related settings.

## Data Availability

The raw data supporting the conclusions of this article will be made available by the authors, without undue reservation.
